# Mitophagy in Hepatic Ischemia–Reperfusion Injury: From Mitochondrial Dysfunction to Therapeutic Targeting

**DOI:** 10.3390/biom16070941

**Published:** 2026-06-24

**Authors:** Xinlei Zou, Tianjie Zhang, Nan Wang, Yuanyue Li, Xingming Jiang, Xiangyu Zhong

**Affiliations:** General Surgery Department, The 2nd Affiliated Hospital of Harbin Medical University, Harbin 150001, China

**Keywords:** mitochondria, mitophagy, hepatic ischemia–reperfusion injury, therapy

## Abstract

Hepatic ischemia–reperfusion injury (HIRI) is a major cause of postoperative liver dysfunction and adverse outcomes in hepatectomy, liver transplantation, and hemorrhagic shock. Among the multiple mechanisms implicated in HIRI, mitochondria are recognized as central organelles that integrate metabolic failure, oxidative stress, inflammation, and cell death. During ischemia, interruption of oxygen and nutrient supply impairs oxidative phosphorylation, depletes ATP, disrupts ionic homeostasis, and renders mitochondria highly vulnerable to subsequent injury. Upon reperfusion, reoxygenation triggers excessive reactive oxygen species production, calcium overload, mitochondrial permeability transition pore opening, and release of damage-associated molecular patterns, thereby amplifying hepatocellular injury and sterile inflammatory responses. As a key component of mitochondrial quality control, mitophagy plays a context-dependent role in HIRI. Appropriate activation of mitophagy facilitates the clearance of damaged mitochondria, limits oxidative stress, restrains inflammasome activation, and preserves hepatocellular homeostasis, whereas insufficient or dysregulated mitophagy contributes to mitochondrial accumulation and aggravates liver injury. This review summarizes mitochondrial alterations during the ischemic and reperfusion phases, outlines the major mitophagy pathways involved in HIRI and discusses recent advances in upstream regulation, disease-specific dysregulation, and mitophagy-targeted interventions. A better understanding of the dynamic and biphasic nature of mitophagy in HIRI may provide a stronger theoretical basis for precision liver-protective strategies and future translational therapies.

## 1. Introduction

Hepatic ischemia–reperfusion injury (HIRI) is commonly encountered in clinical settings such as hepatectomy, liver transplantation, traumatic hemorrhage, and resuscitation after hemorrhagic shock [1,2]. HIRI is not merely the consequence of interrupted blood flow; it is a continuous pathological process initiated by ischemia-induced metabolic disequilibrium, disruption of ionic homeostasis, and organelle injury, and subsequently exacerbated upon reperfusion by reoxygenation, a surge in oxidative stress, and amplification of inflammatory responses [3,4,5]. HIRI profoundly impairs perioperative recovery of liver function, increases postoperative complications, and worsens long term outcomes, making it a major challenge in hepatic surgery and transplant medicine [6].

Given the central role of mitochondria in hepatocellular energy metabolism and stress responses, mitochondrial injury is widely regarded as one of the pivotal events in the initiation and progression of HIRI [7,8]. In addition to supplying ATP through oxidative phosphorylation to sustain normal cellular metabolism, mitochondria are broadly involved in the regulation of ionic homeostasis, clearance of reactive oxygen species (ROS), lipid metabolism, and cell death [9,10,11]. During ischemia, insufficient oxygen and nutrient supply disrupt the mitochondrial electron transport chain, reduce mitochondrial membrane potential, and impair ATP synthesis [12]. Upon reperfusion, although the restoration of oxygen delivery may transiently improve certain aspects of mitochondrial function, the concomitant burst of ROS production and further disturbance of ionic homeostasis instead aggravate mitochondrial dysfunction and cellular injury [13,14]. Thus, mitochondria are not only among the earliest organelles affected during HIRI, but also a critical pathological hub that drives the amplification of subsequent injury.

In recent years, with the deepening understanding of mitochondria, it has become increasingly clear that mitochondrial abnormalities in HIRI extend beyond structural disruption and functional impairment, encompassing multilayered disturbances in mitochondrial quality control, dynamic homeostasis, and organelle adaptive regulation [15,16]. Among these processes, mitophagy, as a key process for the selective removal of damaged mitochondria, is an essential component of maintaining mitochondrial homeostasis. Appropriate mitophagy facilitates the clearance of depolarized and dysfunctional mitochondria, limits persistent ROS generation, suppresses inflammasome activation, and mitigates hepatocyte death. Conversely, insufficient, excessive, or dysregulated mitophagic flux may lead to the accumulation of injured mitochondria and, under certain conditions, even exacerbate cellular damage and inflammatory responses [17,18].

Previous discussions of mitophagy in HIRI have often focused on individual signaling pathways or protective interventions. In contrast, this review adopts an integrated and critical perspective by placing mitophagy within the broader context of liver-specific mitochondrial physiology, phase-dependent mitochondrial injury, metabolic reprogramming, disease-specific hepatic backgrounds, and translational limitations. We first summarize the baseline heterogeneity of hepatic mitochondria and the dynamic mitochondrial alterations that occur during ischemia and reperfusion. We then outline the major ubiquitin-dependent, receptor-dependent, and non-canonical mitophagy pathways involved in HIRI, followed by recent advances in upstream regulation, pathological dysregulation, and mitophagy-targeted interventions. By emphasizing the biphasic and context-dependent nature of mitophagy, this review aims to clarify how mitophagy may be more precisely interpreted and therapeutically modulated in HIRI.

## 2. Mitochondria and HIRI

Mitochondria are central organelles governing hepatocellular energy metabolism and redox homeostasis [19]; accordingly, they occupy a pivotal position in the initiation and progression of HIRI. HIRI is not a single-phase injury event, but rather a dynamic pathological process shaped jointly by the energy crisis of the ischemic phase and the amplification of injury during reperfusion [20,21]. Within this process, mitochondria are not only among the earliest organelles to be affected, but also a major pathological hub driving subsequent oxidative stress, inflammatory escalation, and cell death [22].

### 2.1. Tissue-Specific Features and Baseline Heterogeneity of Hepatic Mitochondria

Mitochondria across tissues do not operate at a uniform basal functional state; instead, they establish distinct oxidative phosphorylation (OXPHOS) baselines shaped by tissue-specific energy demands, substrate preferences, and metabolic roles [23,24]. Compared with heart and skeletal muscle, where mitochondria primarily sustain continuous or rapidly adjustable ATP production, hepatic mitochondria generally display lower overall OXPHOS capacity, mitochondrial content, and mitochondrial DNA (mtDNA) copy number [25,26]. This, however, does not simply denote a “low-activity” mitochondrial phenotype; rather, it reflects a specialized regulatory mode intimately coupled to the liver’s unique metabolic functions. Tissue-specific OXPHOS capacity is governed by mitochondrial abundance, respiratory chain complex content, and the intrinsic activities of individual complexes [27]. Although cardiac mitochondria possess higher OXPHOS complex abundance, hepatic mitochondria sustain a relatively high and stable metabolic state at rest [28]. Notably, certain OXPHOS complexes in hepatic mitochondria, especially complexes IV and V, exhibit relatively high per-complex activity [29,30]. Thus, hepatic mitochondria meet basal metabolic demands not by increasing total OXPHOS complex abundance, but through enhanced per-complex activity and context-dependent metabolic adaptation [31]. Furthermore, the relatively high hepatic cytochrome c oxidase (COX)/citrate synthase (CS) ratio stems largely from low citrate synthase activity, cautioning against the uncritical use of citrate synthase as a universal normalization marker for mitochondrial content across tissues [32,33]. Hence, hepatic respiratory chain activity must be evaluated within the liver’s specific metabolic context.

Metabolically, the hallmark of hepatic mitochondria extends beyond ATP generation to their integration into liver-specific metabolic networks—gluconeogenesis, amino acid metabolism, and lipid metabolism [34,35]. They are enriched in proteins of the urea cycle, lipid metabolism, and amino acid metabolism [36,37]. Moreover, hepatic mitochondria exhibit distinct substrate preferences [38]. Unlike skeletal muscle mitochondria, which rely heavily on complex I-linked substrates for efficient ATP production, hepatic mitochondria display strong capacity for fatty acid metabolism and succinate-supported respiration, while also diverting substrate flux into biosynthetic pathways such as gluconeogenesis and ketogenesis [39,40].

Notably, intrahepatic mitochondria are spatially heterogeneous [41]. Along the periportal–pericentral axis of the hepatic lobule, periportal mitochondria display higher membrane potential, oxygen consumption, maximal respiratory capacity, ATP levels, and mtDNA copy number, and are linked to enhanced fatty acid β-oxidation and OXPHOS activity [42]. Conversely, pericentral mitochondria are associated with lipid synthesis, elevated citrate synthase activity, triglyceride accumulation, and increased mitophagy flux [42]. This zonated heterogeneity, governed by nutrient-sensing signals and protein phosphorylation, underscores the dynamic plasticity and region-specific functional specialization of hepatic mitochondria even under basal conditions [43]. Hence, any discussion of mitochondrial injury, mitophagy, and energy metabolism disturbances in HIRI must first acknowledge the baseline tissue specificity of liver mitochondria [27]. Liver mitochondria are defined by relatively lower overall OXPHOS capacity and mtDNA abundance, yet they possess strong substrate adaptability, regulatory flexibility of respiratory complex activity, metabolic buffering capacity, and spatially organized functional heterogeneity. Collectively, these features may dictate why hepatocytes exhibit patterns of vulnerability and resilience to ischemia-induced hypoxia, reperfusion-associated oxidative stress, and metabolic substrate remodeling that are fundamentally distinct from those in other tissues.

### 2.2. Mitochondrial Alterations During the Ischemic Phase

Ischemia represents the initiating phase of HIRI and the stage during which mitochondrial dysfunction first emerges [44]. Following interruption of hepatic blood flow, the supply of oxygen and metabolic substrates rapidly declines, leading to reduced activity of the mitochondrial electron transport chain (ETC), impaired oxidative phosphorylation, and a marked decrease in ATP production, thereby forcing cells to shift from aerobic metabolism to anaerobic glycolysis [45,46]. However, these early alterations should not be interpreted simply as a nonspecific suppression of the electron transport chain. Because most mitochondrial proteins, including most OXPHOS subunits, are encoded by the nuclear genome, whereas mtDNA encodes only 13 core respiratory-chain polypeptides, HIRI-induced respiratory dysfunction is more appropriately understood as a disruption of nuclear–mitochondrial coordination [47,48].

In this context, Complex I is particularly vulnerable because it contains numerous nuclear-encoded NADH dehydrogenase subunits and Fe–S-containing electron-transfer modules [49,50]. During ischemic stress, downregulation, oxidative modification, or defective assembly of these components can impair NADH oxidation, increase electron leakage, and promote ROS generation [51]. Complex II, also known as succinate dehydrogenase, is entirely nuclear encoded and serves as a critical metabolic interface between the tricarboxylic acid cycle and the respiratory chain [52,53]. During ischemia, altered TCA-cycle flux favors succinate accumulation [54]. Upon reperfusion, rapid oxidation of accumulated succinate through Complex II can drive reverse electron transport at Complex I, thereby amplifying mitochondrial ROS production [55]. Therefore, the ischemic phase not only suppresses mitochondrial respiration but also establishes a reduced and metabolically unstable state that predisposes mitochondria to oxidative injury during subsequent reoxygenation [56].

ATP synthase dysfunction should likewise be viewed as more than a passive consequence of ATP depletion. Proper assembly of Complex V requires the coordinated expression, import, and integration of nuclear-encoded ATP5 subunits, mtDNA-encoded ATP6/ATP8, and multiple assembly factors [57,58]. Loss of mitochondrial membrane potential, impaired protein import, oxidative injury, and disruption of cristae architecture during HIRI may interfere with ATP synthase assembly and oligomerization, thereby further aggravating energetic failure [59].

At the molecular level, ischemia-induced ETC dysfunction is also closely associated with disruption of mitochondrial cofactors and metal-containing prosthetic groups. Fe–S clusters are essential electron-transfer cofactors in Complex I, Complex II, Complex III, and several tricarboxylic acid cycle enzymes, including aconitase [60]. Under ischemic conditions, oxygen deprivation, acidosis, and early oxidative stress may destabilize iron–sulfur (Fe–S)-containing proteins, thereby impairing electron transfer and increasing the probability of electron leakage [61]. In parallel, limited terminal electron acceptor availability promotes NADH accumulation and over-reduction of the coenzyme Q pool [51]. These redox changes do not immediately represent complete mitochondrial collapse, but they create a highly vulnerable metabolic state in which respiratory complexes, quinone cycling, and matrix dehydrogenases become primed for exaggerated ROS production once oxygen is reintroduced [54,55]. Therefore, ischemic mitochondrial injury should be understood not only as a decline in ATP production, but also as a progressive disturbance of Fe–S clusters, NAD^+^/NADH balance, and quinone redox homeostasis [62]. These bioenergetic and redox disturbances are accompanied by early ROS generation, which further damages respiratory chain complexes and disrupts mitochondrial membrane structure and permeability [63,64]. Mitochondrial DNA, which lacks histone protection and possesses limited repair capacity, is particularly vulnerable to oxidative injury at this stage.

As ATP becomes depleted, energy-dependent transport processes, including those mediated by Na^+^/K^+^-ATPase and Ca^2+^-ATPase, are progressively impaired, resulting in gradual disruption of intracellular ionic homeostasis [65,66]. This is manifested by cytosolic acidosis caused by the accumulation of lactate and H^+^, as well as increased Ca^2+^ influx and abnormal mitochondrial Ca^2+^ uptake [67,68]. Increased mitochondrial Ca^2+^ loading not only promotes membrane lipid peroxidation, but also further compromises electron transport chain function, thereby creating a vicious cycle in which impaired energy metabolism and membrane injury mutually reinforce one another [69]. Meanwhile, the propensity for mitochondrial permeability transition pore (mPTP) opening is increased [70]. Although mitochondrial homeostasis has not yet undergone complete collapse at this stage, mitochondrial tolerance to pH fluctuations, ionic disturbances, and subsequent reoxygenation stimuli is already markedly reduced. In parallel, the mitochondrial quality control network also begins to deteriorate during ischemia [71]. Under physiological conditions, appropriate autophagy and mitophagy help eliminate mitochondria damaged at an early stage; however, prolonged ischemia-induced ATP deficiency limits these energy dependent processes, resulting in the progressive accumulation of dysfunctional mitochondria [72,73].

Overall, the mitochondrial alterations during the ischemic phase include suppression of the electron transport chain, ATP depletion, disruption of ionic homeostasis, and heightened vulnerability to injury. Although severe structural damage may not yet have fully developed at this stage, these early mitochondrial disturbances establish the basis for injury amplification during reperfusion.

### 2.3. Mitochondrial Alterations During the Reperfusion Phase

Whereas the ischemic phase is characterized primarily by restricted mitochondrial function and the establishment of injury susceptibility, the reperfusion phase represents a critical period during which mitochondrial damage is rapidly amplified and cell fate is ultimately determined [74,75]. Following restoration of blood flow, oxygen and metabolic substrates are resupplied, allowing transient recovery of mitochondrial electron transport chain activity and ATP-generating capacity [76]. Nevertheless, the re-entry of oxygen and substrates does not simply restore physiological mitochondrial metabolism. Instead, reperfusion imposes a sudden metabolic burden on mitochondria that have already accumulated, reducing equivalents and metabolic intermediates during ischemia [77]. Rapid oxidation of accumulated succinate and NADH promotes over-reduction of the quinone pool and favors electron leakage from the respiratory chain [78]. In parallel, reperfusion in steatotic or metabolically compromised livers may exacerbate mitochondrial fatty-acid handling defects, reflected by impaired β-oxidation, altered acylcarnitine profiles, and lipotoxic stress [79,80]. These changes indicate that reperfusion is characterized not only by reoxygenation injury, but also by maladaptive remodeling of carbohydrate and lipid metabolism within the mitochondrial matrix [81]. Rather than fully reversing injury, this apparent recovery exposes already vulnerable mitochondria to the intense oxidative stress triggered by reoxygenation [82].

The most prominent feature of early reperfusion is the burst of ROS. With the reintroduction of oxygen, xanthine oxidase, NADPH oxidase, the mitochondrial electron transport chain, and uncoupled nitric oxide synthase can all generate large amounts of ROS [83,84]. The mitochondrial component of this ROS burst is strongly influenced by the redox state established during ischemia. During ischemia, succinate can accumulate through reversal of succinate dehydrogenase and related metabolic reactions [55]. Upon reperfusion, rapid oxidation of accumulated succinate through Complex II promotes reduction of the quinone pool and can drive reverse electron transport at Complex I, leading to robust superoxide generation [56]. This mechanism provides a more specific explanation for the abrupt increase in mitochondrial ROS during early reperfusion than a general description of ETC inhibition alone. In addition, reperfusion-associated ROS and reactive nitrogen species can further damage Fe–S clusters and heme-containing cytochromes, aggravating electron leakage and respiratory inhibition [85]. Oxidative modification of cardiolipin and Cytochrome C also weakens mitochondrial membrane integrity and facilitates the transition from respiratory dysfunction to apoptotic signaling [86,87]. Thus, the reperfusion-associated ROS burst reflects a coordinated failure of respiratory complexes, Fe–S cluster stability, heme-dependent electron carriers, NAD^+^/NADH balance, and quinone redox cycling [88,89]. Damaged mitochondria are both a major source of ROS and a principal target of oxidative attack. At the same time, mitochondrial Ca^2+^ overload is further exacerbated and, together with ROS, continuously promotes membrane lipid peroxidation, mtDNA damage, and opening of the mPTP [90,91], thereby leading to mitochondrial depolarization, uncoupling of oxidative phosphorylation, and further ATP depletion [92]. Once mitochondrial function collapses, cell death programs are activated. When a certain level of intracellular ATP is still preserved, the release of pro-apoptotic factors such as Cytochrome C can trigger caspase-dependent apoptosis [93,94]. When ATP depletion becomes more profound, cells are more likely to undergo necrosis [95]. In addition, injured mitochondria can release damage-associated molecular patterns (DAMPs) [96], including mtDNA and mitochondrial proteins, which activate Kupffer cells through signaling pathways such as TLR4/MyD88, TLR9/MyD88, and NF-κB, thereby amplifying the inflammatory response [97,98,99]. In turn, this promotes the recruitment and activation of neutrophils, further aggravating local tissue injury [100]. Thus, during reperfusion, mitochondria are not only targets of injury, but also key organelles linking metabolic dysfunction, cell death, and inflammatory responses [101,102].

Mitochondrial quality control during reperfusion exhibits a clear biphasic nature. The transient restoration of ATP may provide partial support for the reactivation of autophagy and mitophagy, thereby facilitating the clearance of abnormal proteins and damaged mitochondria accumulated during ischemia [103]. Under conditions of severe or sustained injury, however, mitophagic flux is often insufficient to cope with the massive burden of damaged mitochondria, ultimately resulting in loss of quality control [104]. Meanwhile, oxidative stress can induce upregulation of DRP1 and promote mitochondrial fission, whereas downregulation of fusion related proteins such as mitofusin 1 (Mfn1) and Opa1 further disrupts mitochondrial dynamic homeostasis [105,106]. Notably, nitric oxide (NO) signaling during reperfusion exerts bidirectional effects on mitochondria; moderate levels of endothelial nitric oxide synthase (eNOS)-derived NO help improve microcirculation and stabilize mitochondrial function [107,108], whereas excessive NO in an inflammatory milieu can react with superoxide anions to generate peroxynitrite, thereby exacerbating respiratory inhibition and oxidative injury [109].

**Figure 1:**
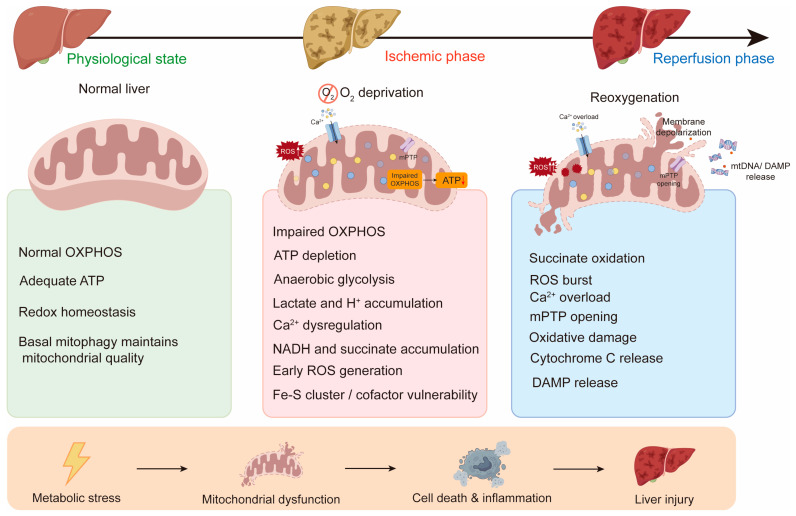
Dynamic mitochondrial changes during the time course of hepatic ischemia–reperfusion injury.

Overall, the defining mitochondrial alterations during the reperfusion phase include the outbreak of oxidative stress, persistent opening of the mPTP, dysregulation of mitochondrial quality control, and amplification of inflammation. In contrast to the ischemic phase, mitochondrial abnormalities at this stage are no longer confined to functional restriction, but instead evolve into structural destruction, cell death, and the escalation of inflammatory cascades. Reperfusion therefore constitutes the decisive stage at which HIRI progresses from reversible metabolic disturbance to irreversible tissue injury (Figure 1).

## 3. Mitophagy Pathways

Mitophagy is a highly selective form of autophagy that plays a central role in mitochondrial quality control by recognizing and eliminating damaged or dysfunctional mitochondria [110]. Research into the regulatory mechanisms of mitophagy in HIRI has advanced substantially in recent years. Current evidence indicates that mitophagy is not a unidirectional protective mechanism, but rather a dynamic process jointly shaped by the duration of ischemia, the intensity of reperfusion, the stability of mitochondrial membrane potential, the level of oxidative stress, and the inflammatory microenvironment. Restoration or enhancement of mitophagy within an appropriate temporal window facilitates the clearance of dysfunctional mitochondria, preserves mitochondrial network homeostasis, and alleviates tissue injury. Therefore, understanding the distinct molecular features and functional interplay of these pathways is essential for clarifying how mitophagy is initiated, amplified, and regulated during HIRI.

### 3.1. Ubiquitin-Dependent Mitophagy

The PINK1/Parkin axis remains the most classical and mechanistically best characterized ubiquitin-dependent pathway of mitophagy [111]. Under conditions of intact mitochondrial function, PTEN-induced kinase 1 (PINK1) is imported into mitochondria through the coordinated action of the translocase of the outer membrane and translocase of the inner membrane complexes [112], and is subsequently processed sequentially by inner membrane-associated proteases such as mitochondrial processing peptidase and presenilin-associated rhomboid-like protein (PARL), ultimately being released into the cytosol for degradation [113,114,115]. By contrast, when mitochondria undergo depolarization or defects in protein import, PINK1 becomes stabilized and accumulates on the outer mitochondrial membrane [116,117]. Accumulated PINK1 phosphorylates ubiquitin on outer mitochondrial membrane proteins as well as Parkin itself, thereby relieving Parkin autoinhibition and enhancing its E3 ubiquitin ligase activity [118]. This in turn promotes the ubiquitination of multiple outer mitochondrial membrane proteins, including Mfn1/2, dynamin related protein 1 (DRP1), MIRO1/2, and voltage-dependent anion channel 1/2, resulting in the progressive amplification of phosphorylated ubiquitin chain signals on the surface of damaged mitochondria [119,120].

On this basis, ubiquitin-binding autophagy receptors, including OPTN, NDP52, TAX1BP1, p62, and NBR1, are recruited to the damaged mitochondrial surface [121,122,123]. Through their ubiquitin-binding domains and LC3 interacting regions, these receptors link ubiquitin-labeled mitochondria to the autophagic membrane, thereby promoting autophagosome formation and subsequent lysosomal degradation [124,125]. Among these receptors, OPTN and NDP52 appear to play particularly critical roles in mitophagy, whereas p62 and NBR1 function more prominently as accessory substrates that contribute to pathway amplification [126,127]. At the same time, outer mitochondrial membrane deubiquitinases such as USP30 antagonize the PINK1/Parkin pathway by removing ubiquitin chains from the mitochondrial surface, indicating that ubiquitin-dependent mitophagy is not a process of simple one-way activation, but rather is maintained in a state of continuous dynamic equilibrium [128].

### 3.2. Receptor-Dependent Mitophagy

In addition to the PINK1/Parkin-mediated ubiquitin-dependent pathway, receptor-dependent mitophagy also constitutes a major mechanism for maintaining mitochondrial quality control. A defining feature of this class of pathways is that receptor proteins located on the mitochondrial membrane directly bind members of the LC3/GABARAP family through their own LC3 interacting regions (LIRs), thereby promoting the direct entry of mitochondria into the autophagic pathway [129].

BNIP3 and NIX remain the most representative molecules in hypoxia-associated receptor-dependent mitophagy [130,131,132]. Both are transcriptionally upregulated under the control of hypoxia inducible factor 1α, while also being subject to multiple layers of post translational regulation. Phosphorylation of BNIP3 at Ser17 and Ser24 [130], as well as of NIX at Ser34 and Ser35 [133], enhances their binding affinity for ATG8 family proteins, a process that can be directly promoted by ULK1 [134,135,136]. At the same time, JNK1/2 stabilizes BNIP3 through phosphorylation [137], whereas the FBXL4/PPTC7-associated complex promotes the degradation of BNIP3 and NIX, thereby forming a regulatory circuit that contributes to mitochondrial homeostasis [138,139]. Importantly, BNIP3/NIX signaling is not entirely independent of the PINK1/Parkin pathway. BNIP3 can interact with PINK1 and influence its proteolytic processing, whereas NIX can be ubiquitinated by Parkin and subsequently recruit NBR1, indicating substantial crosstalk between receptor-dependent and ubiquitin-dependent mitophagy [126]. FUNDC1 is another key protective receptor under hypoxic and stress conditions [140]. In addition to functioning as an LC3 binding receptor, FUNDC1 is deeply involved in the regulation of mitochondrial dynamics [141]. Under conditions of mitochondrial stress, the interaction between FUNDC1 and the fusion-related protein OPA1 is weakened, whereas its association with the fission protein DRP1 is enhanced, thereby promoting mitochondrial fission, and creating the structural basis for subsequent mitophagy [142]. This process is tightly controlled through phosphorylation at key residues of FUNDC1. In addition, FUNDC1 itself is subject to ubiquitination, and alterations in its stability help determine the activation threshold of receptor-mediated mitophagy [143].

Beyond these classical receptors, several non-canonical receptors have also been identified as important contributors to mitophagy. BCL2L13 is regarded as the mammalian functional counterpart of the yeast mitophagy receptor ATG32 [144,145]. Under Parkin-independent conditions, it can recruit ULK1 and LC3B to form a BCL2L13/ULK1/LC3B complex, while also promoting mitochondrial fission and clearance through regulation of DRP1 phosphorylation [146,147]. FKBP8 participates in mitophagy by binding LC3A through its LIR-like motif [148], and, during this process, translocates from mitochondria to the endoplasmic reticulum to avoid its own degradation [149]. AMBRA1 functions both as a regulator of autophagy and as a mitophagy receptor [150]. It can enhance PINK1 stability and promote Parkin-dependent mitophagy, while also independently driving mitochondrial clearance in the absence of Parkin or PINK1 [151].

### 3.3. Other Pathways

Recent studies suggest that certain components of the mitochondrial inner membrane can also function as mitophagy receptors after rupture of the outer mitochondrial membrane. Among these, prohibitin 2 (PHB2) is the most representative example [152]. In the presence of Parkin, mitochondrial injury induced by carbonyl cyanide 3-chlorophenylhydrazone (CCCP) or by oligomycin/antimycin A can cause rupture of the outer mitochondrial membrane, thereby exposing PHB2, which normally resides in the inner membrane [153,154]. Once exposed, PHB2 binds LC3 through its LIR, promoting the contact of autophagic precursor membranes with the damaged inner mitochondrial membrane and facilitating mitochondrial sequestration. Loss of PHB2 or mutation of its LIR markedly impairs damage-induced mitophagy. Beyond serving as a receptor, PHB2 can also stabilize PINK1 by negatively regulating PARL, indicating that it is not only an effector receptor but also an important auxiliary regulator of PINK1 homeostasis [155].

In contrast to conventional protein receptors, certain mitochondrial membrane lipids can themselves act as autophagic signals under conditions of stress or damage. The prototypical molecule in this context is cardiolipin [156]. Cardiolipin is mainly localized to the inner mitochondrial membrane and to contact sites between the inner and outer membranes [157], where it supports the assembly of electron transport chain complexes and the stability of crista architecture [158]. Upon mitochondrial injury, cardiolipin can translocate to the outer mitochondrial membrane and bind LC3, particularly LC3A, thereby directly promoting recognition of damaged mitochondria by the autophagic machinery [159]. Inhibition of cardiolipin synthesis, disruption of its translocation, or interference with its remodeling all impair mitophagy. Proteins such as Beclin 1 can also associate with cardiolipin-enriched membranes and promote membrane curvature, further supporting an active role for lipid signaling in autophagosomal membrane formation [160,161]. In addition to cardiolipin, ceramide has likewise emerged as part of the lipid-mediated mitophagy repertoire. C18-ceramide can promote LC3B lipidation and direct autophagosomes toward mitochondria through a mechanism dependent on mitochondrial fission and ceramide synthase 1-related signaling [162]. Notably, this pathway exhibits marked bidirectionality across physiological and pathological contexts; in the maintenance of tissue homeostasis during aging, moderate ceramide mediated mitophagy may exert compensatory and protective effects, whereas in certain tumor cells it may instead trigger lethal autophagic elimination [163,164].

Another important mode of mitochondrial quality control is piecemeal mitophagy. Rather than engulfing an entire mitochondrion, this process selectively degrades individual mitochondrial proteins, protein complexes, or even damaged mtDNA. Unlike canonical mitophagy, piecemeal mitophagy can occur in the absence of overt mitochondrial depolarization and does not require Parkin overexpression. Proteins such as SAMM50 and MTX1, components of the sorting and assembly machinery complex, as well as IMMT and MIC60, a component of the mitochondrial contact site and cristae organizing system [165,166], can serve as autophagic cargoes and undergo selective degradation through interactions with ATG8 family proteins in a p62-dependent manner [167]. By contrast, SNX10 acts as a negative regulator of this process under hypoxia mimetic conditions, limiting the piecemeal clearance of certain oxidative phosphorylation components [168,169]. More notably, the “piecemeal” targets of mitophagy are not restricted to proteins. Under conditions of oxidative stress or mtDNA damage, the primate-specific protein ATAD3B mediates the selective elimination of damaged mtDNA. Mechanistically, mtDNA injury promotes dissociation of the ATAD3B/ATAD3A complex, exposing the C terminus of ATAD3B on the outer mitochondrial membrane and enabling recruitment of LC3 [170,171].

Taken together, mitophagy is not governed by a single dominant pathway, but instead comprises a multilayered and dynamically coupled network shaped by hypoxic stress, alterations in mitochondrial membrane architecture, the inflammatory milieu, and the metabolic state of the cell.

## 4. Advances in the Regulation of Mitophagy in HIRI

As understanding of mitochondrial homeostatic imbalance in HIRI has deepened, accumulating evidence indicates that mitophagy does not occur in isolation, but is instead subject to fine regulation by multiple upstream signaling molecules and stress-responsive pathways. These regulatory factors can influence the clearance of damaged mitochondria through the canonical PINK1/Parkin pathway, while also modulating mitochondrial quality control through receptor-dependent and other non-canonical mechanisms.

### 4.1. Regulation of PINK1/Parkin Pathway

A growing number of studies have focused on the upstream regulatory mechanisms governing PINK1/Parkin-mediated mitophagy, suggesting that this classical pathway is influenced by inflammation, endoplasmic reticulum stress, transcriptional control, and programmed cell death.

Proprotein convertase subtilisin/kexin type 9 (PCSK9), synthesized by hepatocytes, has been shown to be markedly upregulated during HIRI. Further studies indicate that PCSK9 suppresses PINK1/Parkin-mediated mitophagy, thereby activating STING/NLRP3-associated inflammatory signaling and aggravating HIRI. Consistently, inhibition of hepatocellular PCSK9 expression promotes PINK1/Parkin-dependent mitophagy, reduces inflammatory responses induced by ROS accumulation and mtDNA release, and consequently exerts hepatoprotective effects [172]. DJ-1, a molecule closely linked to oxidative stress, has likewise been implicated in the regulation of the PINK1/Parkin pathway in HIRI. One study showed that HIRI leads to downregulation of DJ-1 expression in liver tissue, whereas hepatocyte-specific DJ-1 deficiency unexpectedly and significantly attenuates HIRI-induced liver injury and inflammatory responses. This protective effect depends on the enhancement of PINK1/Parkin-mediated mitophagy caused by DJ-1 deficiency because the hepatoprotective effect conferred by DJ-1 knockout is reversed when autophagy is inhibited with 3-methyladenine or chloroquine. These findings suggest that aberrant alterations in DJ-1 during HIRI regulate mitochondrial quality control through modulation of the PINK1/Parkin axis [173].

In addition to these molecules, the necroptosis-related effector mixed lineage kinase domain-like protein (MLKL) has also been shown to be closely associated with PINK1-mediated mitophagy. Studies have demonstrated that ischemia–reperfusion stimulation upregulates MLKL expression in the livers of wild type mice, whereas MLKL deficiency alleviates HIRI and suppresses activation of the cGAS-STING signaling pathway in hepatic macrophages; notably, this protective effect is abolished by administration of a STING agonist. Moreover, MLKL deficiency enhances PINK1-mediated mitophagy, thereby helping to attenuate oxidative DNA damage in hepatocytes. These findings suggest that MLKL may participate in the pathogenesis of HIRI by regulating PINK1-dependent mitophagy and its associated inflammatory signaling pathways [174]. X box binding protein 1 (XBP1), an endoplasmic reticulum stress-related transcription factor [175], is likewise considered an important upstream suppressor of the PINK1/Parkin pathway. HIRI induces hepatocellular injury accompanied by high XBP1 expression, whereas hepatocyte-specific deletion of XBP1 alleviates hepatocyte injury and necrosis and increases the expression of mitophagy-related markers, thereby mitigating HIRI. Mechanistically, XBP1 can directly interact with FOXO1 and promote its ubiquitination and proteasomal degradation. Targeting XBP1, by contrast, helps restore FOXO1 expression, enhances PINK1/Parkin pathway activity, and thereby promotes mitophagy and exerts hepatoprotective effects [176].

PINK1-mediated mitophagy is closely linked to the regulation of innate immune inflammation in the liver. Kupffer cells highly express NLRP3, and hepatic ischemia–reperfusion is commonly accompanied by marked hepatic inflammation, activation of the NLRP3 inflammasome, and enhancement of PINK1-mediated mitophagy [177]. It has been shown that mitophagy augmentation driven by PINK1 overexpression further suppresses NLRP3 activation and reverses Kupffer cell-mediated inflammatory injury in hepatocytes. By contrast, this protective effect is completely abolished by either a kinase inactive PINK1 mutant or different autophagy inhibitors. These findings indicate that PINK1-mediated mitophagy is of central importance for restraining NLRP3 inflammatory signaling during HIRI [178]. The augmenter of liver regeneration (ALR) protein, encoded by the growth factor erv1-like gene, is highly expressed in the liver, and is predominantly localized in the cytoplasm, nucleus, and mitochondria [179]. Its role in mitophagy is likewise critical, as its depletion leads to rapid ATP loss and consequent cell death. In addition, ALR can restore defective mitophagy by upregulating Mfn2, thereby promoting PINK1 accumulation and Parkin translocation to mitochondria; conversely, Mfn2 deficiency weakens this protective process [180].

Overall, current evidence suggests that the PINK1/Parkin pathway is not merely a simple mitochondrial damage response module, but rather an interactive signaling hub jointly regulated by metabolic modulators, endoplasmic reticulum stress, programmed cell death, and inflammatory signaling networks in HIRI.

### 4.2. Regulation of Non-Canonical Mitophagy

Beyond the canonical PINK1/Parkin pathway, multiple additional mechanisms regulate mitophagy in HIRI, involving receptor-dependent pathways, post transcriptional regulation, and signaling networks closely linked to mitochondrial dynamics.

In HIRI models, the expression of NIPSNAP1 and insulin-like growth factor 2 mRNA binding protein 2 (IGF2BP2) is markedly downregulated. As a receptor-associated molecule, NIPSNAP1 exerts a positive regulatory effect on mitophagy; its knockdown suppresses mitophagy, whereas its overexpression produces the opposite effect [181]. Mechanistically, IGF2BP2, acting as an m6A reader, directly binds NIPSNAP1 transcripts and regulates their mRNA stability, thereby promoting mitophagy and preserving mitochondrial homeostasis. These findings suggest that epitranscriptomic regulation may play an important role in HIRI-associated mitophagy control [182].

CCAAT/enhancer binding protein homologous protein (CHOP) is a key regulator of cellular stress responses and cell death [183]. Studies have shown that mice with hepatocyte-specific CHOP deficiency exhibit reduced cell death, lower ROS production, and enhanced mitophagy following ischemia–reperfusion. Further mechanistic analyses indicate that CHOP deficiency promotes mitophagy by upregulating DRP1 and enhancing Beclin 1 activation [184].

Phosphoglycerate mutase family member 5 (PGAM5), a mitochondria resident phosphatase, has recently emerged as an important regulator of mitophagy, mitochondrial fission, mitochondrial biogenesis, and multiple forms of cell death [185]. In HIRI, miR-330-3p can regulate mitophagy through its interaction with PGAM5. Overexpression of miR-330-3p reduces PGAM5 levels, thereby suppressing mitophagy during ischemia–reperfusion injury; conversely, downregulation of miR-330-3p is associated with increased PGAM5 expression and consequent enhancement of mitophagy [186]. In addition, the study by Hong et al. further suggests that the protective effect of heme oxygenase 1 (HO-1) extends beyond antioxidative and anti-inflammatory actions and is also closely linked to the maintenance of mitochondrial homeostasis. HO-1 ameliorates mitochondrial dysfunction under ischemia–reperfusion stress by modulating PGAM5 associated mitochondrial quality control processes, thereby attenuating HIRI-induced injury [187]. Collectively, these findings identify PGAM5 as a key node linking mitophagy, mitochondrial dynamics, and cell injury.

Taken together, mitophagy in HIRI is regulated not only by the classical PINK1/Parkin axis, but also by receptor-associated molecules, m6A-dependent post transcriptional regulation, stress-responsive transcription factors, and PGAM5-related mitochondrial quality control networks, underscoring the multilayered and highly complex nature of its regulation (Table 1).

## 5. Dysregulation of Mitophagy Under Specific Pathological Conditions

In clinical settings, HIRI rarely occurs in the context of a completely normal liver, and is frequently superimposed on underlying conditions such as aging, diabetes, and fatty liver disease [188]. These pathological backgrounds can further reshape the mitophagic response, thereby influencing both susceptibility to hepatic ischemia–reperfusion injury and the capacity for post injury repair [189].

The regenerative capacity of the aged liver declines with advancing age, and recovery from HIRI is correspondingly impaired, a phenomenon closely associated with reduced mitophagy. Compared with young hepatocytes, aged livers exhibit an almost complete loss of SIRT1 and Mfn2 after ischemia–reperfusion. Notably, overexpression of either SIRT1 or Mfn2 alone is insufficient to attenuate HIRI, whereas simultaneous overexpression of both promotes autophagy and prevents post reperfusion mitochondrial dysfunction and cell death. Mechanistically, SIRT1 deacetylates the C terminal lysine residues K655 and K662 of Mfn2, thereby inducing autophagic activation. These findings indicate that the SIRT1/Mfn2 axis is critical for the maintenance of mitophagy in the aged liver [190,191]. In addition, reduced phosphorylation of the endoplasmic reticulum stress-responsive factor eIF2α in aged livers has also been identified as an important cause of impaired Parkin-dependent mitophagy. Studies have shown that decreased eIF2α phosphorylation leads to downregulation of Parkin and Atg5. eIF2α knockdown increases mitochondrial ROS production and reduces LC3B recruitment to mitochondria, suggesting that eIF2α activation contributes to the maintenance of Parkin-dependent mitophagy. Furthermore, pharmacological maintenance of eIF2α phosphorylation with salubrinal not only restores Parkin expression and promotes mitochondrial clearance in aged mouse livers, but also reduces mitochondrial ROS production and apoptosis, thereby ameliorating HIRI [192].

Diabetes likewise represents an important pathological background influencing the outcome of HIRI [193]. Studies have shown that HIRI is markedly aggravated in diabetic mice and is accompanied by increased mitochondrial oxidative stress; conversely, scavenging mitochondrial ROS alleviates liver injury in diabetic animals [194]. Mechanistic analyses indicate that AMPK-mediated mitophagy is suppressed in diabetic mice after ischemia–reperfusion. Treatment with an AMPK agonist effectively restores mitophagy, thereby reducing mitochondrial oxidative stress and attenuating HIRI in diabetic mice. These findings suggest that defective AMPK-dependent mitophagy may be one of the key mechanisms underlying the heightened severity of HIRI in the diabetic setting [195].

In addition to diabetes, alcohol-associated fatty liver also increases hepatic susceptibility to ischemia–reperfusion injury. Studies have shown that, compared with controls, mice with alcohol-diet-induced steatosis liver disease exhibit higher serum aminotransferase activities, elevated levels of proinflammatory cytokines, and more severe histological injury after reperfusion. 2-Methoxyestradiol (2-ME2), an endogenous metabolite of estradiol with antioxidative and anti-inflammatory properties, markedly ameliorates these abnormalities. Further investigation revealed that, in the combined alcohol diet plus ischemia–reperfusion model, both autophagy and mitophagy are significantly reduced, accompanied by marked downregulation of the Atg12-Atg5 complex, Atg3, Atg7, lysosome-associated membrane protein 2 (LAMP2), and Rab7 in the liver; all these changes are attenuated by 2-ME2 treatment. In the same model, SIRT1 protein expression and activity are also significantly decreased, whereas 2-ME2 restores SIRT1 levels. These observations suggest that 2-ME2 may alleviate ischemia–reperfusion-induced hepatocellular injury in alcohol-associated fatty liver by activating SIRT1-dependent autophagic signaling [196].

Taken together, pathological states such as aging, diabetes, and fatty liver disease impair mitophagic capacity, aggravate mitochondrial oxidative stress, and exacerbate cellular injury through disruption of autophagy-related signaling pathways involving SIRT1/Mfn2, eIF2α/Parkin, AMPK, and SIRT1. These findings not only identify pre-existing pathological conditions as major determinants of HIRI vulnerability, but also underscore that future mitophagy-targeted interventions should fully consider the heterogeneity of patients’ baseline hepatic status and metabolic background.

## 6. Therapeutic Advances in Targeting Mitophagy in HIRI

As a central component of mitochondrial quality control, mitophagy has increasingly emerged as an important therapeutic entry point in HIRI research. Current evidence broadly supports the view that, during HIRI, mitophagy is a key regulatory mechanism determining whether mitochondrial damage can be removed in a timely manner and whether inflammation and oxidative stress can be effectively contained. Ischemia–reperfusion can impair mitophagy, aggravate mitochondrial fragmentation, and suppress mitochondrial biogenesis, ultimately resulting in the persistent accumulation of damaged mitochondria, Cytochrome C release, hepatocellular apoptosis, and amplification of inflammation. Accordingly, interventions aimed at restoring mitophagy and overall mitochondrial quality control are now regarded as a major direction in the development of potential therapies for HIRI.

### 6.1. Therapeutic Potential of Natural Bioactive Compounds

In recent years, natural bioactive compounds have attracted broad attention for the prevention and treatment of HIRI because of their multi-target properties, relatively low toxicity, and broad biological effects on oxidative stress, inflammatory responses, and mitochondrial homeostasis. Existing studies indicate that a variety of naturally derived compounds can alleviate HIRI by modulating mitophagy, improving mitochondrial quality control, and suppressing related cell death pathways.

#### 6.1.1. Flavonoids and Polyphenolic Compounds

Tangeretin (TAN), a polymethoxylated flavone derived from citrus peel, exhibits prominent antioxidative and anti-inflammatory activities. Studies have shown that TAN enhances multiple biological processes associated with mitochondrial homeostasis, particularly mitophagy and the regulation of ferroptosis. TAN treatment activates mitophagy and reduces intracellular ferrous iron (Fe^2+^) and lipid peroxide (LPO) levels in hepatocytes. Notably, TAN-induced mitophagy decreases the accumulation of mitochondrial Fe^2+^ and LPO, thereby coordinately suppressing ferroptosis. Mechanistic studies further indicate, through molecular docking and dynamic simulation analyses, that TAN displays strong affinity for the Nrf2/Keap1 complex, promotes the nuclear translocation of Nrf2, and subsequently activates mitophagy while inhibiting hepatocellular ferroptosis. Liver-specific knockdown of Nrf2 abolishes the protective effects of TAN, suggesting that Nrf2 may serve as a major molecular basis for its hepatoprotective action [197].

Pterostilbene (Pt), a natural bioactive compound present in blueberries and grapes, has likewise shown potential in the prevention of HIRI. Studies demonstrate that Pt pretreatment ameliorates HIRI by attenuating histopathological damage, reducing hepatocyte apoptosis, and lowering plasma alanine aminotransferase (ALT) and aspartate aminotransferase (AST) levels. In in vitro hypoxia/reoxygenation models, Pt also markedly alleviates the increase in apoptosis, mitochondrial membrane dysfunction, and excessive accumulation of mitochondrial ROS. Mechanistically, Pt effectively enhances mitophagy, whereas its protective effects are significantly weakened after transfection with PINK1 siRNA, indicating that Pt exerts hepatocellular protection primarily through activation of PINK1-dependent mitophagy [198].

Taxifolin (TAX), a natural flavonoid compound, possesses a range of biological activities, including antioxidative, anti-inflammatory, and antitumor effects. Studies have shown that TAX pretreatment improves HIRI by restoring mitochondrial membrane potential in hepatocytes after ischemia–reperfusion and suppressing the expression of pro-apoptotic proteins. Further investigations indicate that TAX exerts its protective effects mainly by activating the PINK1/Parkin pathway and enhancing mitophagy. Consistently, silencing of PINK1 in primary hepatocytes markedly reverses the beneficial effects of TAX, further supporting a mechanism dependent on PINK1/Parkin-mediated mitophagy [199].

Formononetin (FN), an isoflavone primarily isolated from soybean and red clover, has demonstrated anti-inflammatory and antioxidative effects in multiple degenerative and cholestatic diseases. Studies indicate that FN exerts a marked protective effect against HIRI, and that this effect is closely associated with PINK1/Parkin-regulated mitophagy. Specifically, FN promotes activation of the PINK1/Parkin signaling axis by upregulating PHB2 expression, suppressing PARL expression, and preventing PGAM5 cleavage. Further studies show that deletion of either PINK1 or PHB2 abolishes the hepatoprotective effect of FN. These findings suggest that FN may attenuate ischemia–reperfusion-induced liver injury by promoting PHB2/PINK1/Parkin-mediated mitophagy [200].

#### 6.1.2. Terpenoids and Saponins

In addition to flavonoids and polyphenolic compounds, terpenoids, saponins, and other natural bioactive molecules have also demonstrated important therapeutic potential in the intervention of HIRI.

Genipin, an iridoid compound derived from Gardenia jasminoides, has been shown to possess antioxidative and anti-inflammatory properties. In experimental studies, intraperitoneal administration of genipin to mice 1 h before ischemia ameliorated hepatocellular oxidative injury and mitochondrial dysfunction. Further investigations indicated that genipin significantly suppresses the increase in mitochondrial fission induced by hepatic ischemia–reperfusion, while upregulating the expression of proteins associated with mitochondrial biogenesis, mitophagy, and fusion. In addition, the I/R-induced decrease in sirtuin 1 (SIRT1) protein levels and the reduction in AMPK phosphorylation can both be partially reversed by genipin. These findings suggest that genipin may exert hepatoprotective effects by regulating mitochondrial quality control through the SIRT1/AMPK pathway [201].

Importantly, not all studies suggest that enhancing mitophagy is invariably beneficial in HIRI. Under certain conditions, excessive or dysregulated mitophagy may also contribute to the aggravation of hepatocellular injury. Ginsenoside Rg1, a triterpenoid glycoside, provides additional support for this view. Studies have shown that Rg1 alleviates HIRI through modulation of the mitophagy pathway. In vivo experiments demonstrated that Rg1 maintains the stability of mitochondrial membrane potential (MMP) and suppresses the excessive activation of mitophagy and related signaling pathways during hepatic ischemia–reperfusion. In vitro studies likewise confirmed that Rg1 effectively improves cell viability after hypoxia/reoxygenation injury, inhibits apoptosis, and stabilizes MMP. Overall, Rg1 exerts hepatoprotective effects by modulating the PINK1/Parkin signaling pathway and the level of mitophagy. These findings further suggest that mitophagy may exert dual effects in HIRI, with its protective role depending on appropriate regulation rather than excessive activation [202].

#### 6.1.3. Endogenous Lipids and Metabolic Mediators

In addition to exogenous natural bioactive compounds, certain endogenous lipid mediators and metabolic molecules have also been shown to participate in the prevention and treatment of HIRI by regulating mitochondrial quality control and mitophagy, thereby opening new avenues for therapeutic investigation.

Resolvin D1 (RvD1), a specialized pro resolving lipid mediator, possesses both anti-inflammatory and antioxidative activities [203]. Studies have shown that RvD1 intervention significantly attenuates hepatocellular injury in HIRI, restores mitochondrial function, and reduces oxidative damage. Mechanistic analyses further revealed that ischemia–reperfusion leads to downregulation of thioredoxin 2 (TRX2) expression and enhances its interaction with thioredoxin interacting protein (TXNIP), whereas RvD1 mitigates these changes and thereby preserves TRX2 function. Notably, when TRX2 is silenced by siRNA, the protective effects of RvD1 on mitochondrial quality control and hepatocyte survival are markedly weakened. Collectively, these findings indicate that RvD1 alleviates HIRI by regulating TRX2-mediated mitochondrial quality control and thereby improving ischemia–reperfusion-induced oxidative stress and mitochondrial dysfunction [204].

25-Hydroxycholesterol (25-HC), an oxysterol involved in inflammatory and immune regulation, has also been implicated in hepatoprotection during ischemia–reperfusion. Studies have shown that intraperitoneal administration of 25-HC 4 h before ischemia effectively lowers serum aminotransferase levels, reduces apoptosis, and attenuates hepatic tissue injury in mice. Further mechanistic investigations demonstrated that 25-HC pretreatment activates PINK1/Parkin-dependent mitophagy while suppressing activation of the NLRP3 inflammasome. When the mitophagy inhibitor 3 methyladenine (3-MA) is administered, the protective effects of 25-HC against HIRI and NLRP3 inflammasome activation are markedly diminished, suggesting that its hepatoprotective effects depend, at least in part, on mitophagy activation [205].

Nicotinic acid (NA) exerts antioxidative and metabolic regulatory effects and has likewise been considered a promising candidate for ameliorating HIRI. Studies have shown that NA pretreatment lowers serum ALT, AST, and lactate dehydrogenase (LDH) levels; suppresses inflammatory responses by reducing neutrophil infiltration and macrophage activation; and alleviates hepatic oxidative stress by decreasing ROS levels and malondialdehyde levels. Mechanistically, NA confers hepatoprotection by enhancing mitophagy, promoting mitochondrial biogenesis, and preserving the stability of the mPTP [206].

### 6.2. Clinical Pharmacological Interventions

In addition to natural bioactive compounds, several clinically used drugs and their derivative therapeutic strategies have also been shown to alleviate HIRI by regulating mitochondrial homeostasis, suppressing inflammation, and ameliorating cell death processes. These agents may therefore possess greater translational potential.

Dexmedetomidine (Dex) has demonstrated neuroprotective effects in multiple models of brain injury [207]. Studies have shown that, in rat models of HIRI, Dex effectively attenuates neuroinflammation and cognitive impairment by upregulating the expression and activity of sirtuin 3 (SIRT3). At the same time, Dex suppresses activation of the NLRP3 inflammasome, whereas either the SIRT3 inhibitor 3-TYP or the autophagy inhibitor 3-MA abolishes its protective effects. Further investigation revealed that 3-TYP treatment downregulates several downstream targets of SIRT3, including FOXO3α, SOD2, PRDX3, and CYP-D, whereas these proteins are largely unaffected by 3-MA. However, both 3-TYP and 3-MA negate the antioxidative and anti-apoptotic effects of Dex. These findings suggest that Dex exerts protection primarily by suppressing HIRI-induced NLRP3 inflammasome activation through SIRT3-mediated mitophagy [208].

In addition, combination strategies based on drug delivery systems have shown emerging therapeutic promise. One study demonstrated that metformin (Met)-loaded adipose-derived stem cell exosomes (ADSC-Exo) exert superior protective effects against HIRI compared with ADSC-Exo alone. In a miniature pig model of HIRI, metformin-loaded exosomes (Met-Exo) more effectively activated the AMPK/SIRT1 signaling axis, thereby regulating multiple aspects of mitochondrial quality control, including improvement of mitochondrial dynamics, promotion of mitochondrial biogenesis, and suppression of persistent excessive mitophagy after injury. Through restoration of mitochondrial function, Met-Exo further preserved ATP production in liver tissue and reduced ROS accumulation, thereby alleviating mitochondrial dysfunction. Once mitochondrial status was improved, Met-Exo also further suppressed pyroptosis and reduced mitochondrial injury mediated by gasdermin D-N, thus providing dual protection against cellular damage. Ultimately, by inhibiting pyroptosis-associated inflammatory responses, Met-Exo reduced the release of inflammatory mediators such as IL-1β and IL-18 and attenuated hepatic inflammatory injury. These findings indicate that Met-Exo, through the coordinated modulation of mitochondrial dysfunction and pyroptosis, exerts robust tissue protective effects in HIRI and offers a new therapeutic perspective for liver transplantation and HIRI intervention [209].

Overall, clinically used drugs and their derivative delivery strategies exhibit considerable promise in the prevention and treatment of HIRI. Their advantage lies in the ability not only to regulate mitochondrial quality control, but also to simultaneously target multiple critical processes, including inflammation, oxidative stress, and programmed cell death [210].

### 6.3. Non-Pharmacological Interventions

In addition to pharmacological treatment, non-pharmacological preconditioning strategies also represent an important direction in the prevention and treatment of HIRI. These interventions exert protective effects by enhancing the tolerance of cells or tissues to ischemia–reperfusion stress and by strengthening mitochondrial quality control and cellular adaptive capacity [211].

Hypoxic preconditioning can enhance the protective effect of human bone marrow mesenchymal stem cells (hBMSCs) against HIRI, with the core mechanisms being closely related to improved mitochondrial quality and increased intercellular mitochondrial transfer. Studies have shown that hypoxic preconditioning reduces superoxide accumulation in hBMSCs and restores mitochondrial membrane potential through the induction of mitophagy, thereby improving mitochondrial quality. Compared with untreated hBMSCs, hypoxia preconditioned hBMSCs (hypo hBMSCs) more effectively attenuate HIRI. Further mechanistic studies indicate that hypo hBMSCs can transfer a greater number of high-quality mitochondria to hepatocytes through gap junctions (GJs), a process that is enhanced by the GJ agonist retinoic acid and attenuated by the inhibitor Gap26, suggesting a central role for GJs in mitochondrial transfer. At the molecular level, hypoxic preconditioning markedly upregulates the expression of connexin 43 (Cx43) and connexin 32 (Cx32) in hBMSCs. The induced Cx43 and Cx32 then form homotypic Cx43-GJs and Cx32-GJs, respectively, with hepatocytes rather than heterotypic Cx43-Cx32-GJs, thereby enhancing the efficiency of mitochondrial transfer between hBMSCs and hepatocytes. Taken together, hypoxic preconditioning not only improves the intrinsic mitochondrial quality of hBMSCs, but also promotes the transfer of high-quality mitochondria to hepatocytes through Cx43/Cx32-mediated gap junctions, thereby effectively alleviating HIRI. These findings provide a new perspective for the application of hBMSCs in liver protection after transplantation [212].

Remote ischemic preconditioning (RIPC), by contrast, can ameliorate HIRI caused by post traumatic hemorrhagic shock resuscitation (HSR) through enhancement of Parkin-dependent mitophagy [213]. Studies have shown that HSR markedly aggravates hepatocellular injury and hepatic necrosis, accompanied by elevated levels of inflammatory cytokines such as IL-6 and TNF-α. When RIPC is applied before injury onset, however, both hepatic damage and the systemic inflammatory response are significantly attenuated. Importantly, this protective effect is largely lost in Parkin-deficient (Parkin^−/−^) mice, indicating that Parkin is a key mediator of the hepatoprotective action of RIPC. Further mechanistic analyses revealed that RIPC alone is insufficient to robustly induce mitophagy, but when administered before HSR, it acts synergistically with the subsequent injury stimulus to enhance mitophagy and to induce adaptive changes in mitochondrial morphology that favor mitophagic clearance. None of these changes are observed in Parkin^−/−^ mice. These findings indicate that the protective effect of RIPC against post HSR hepatic injury depends on the activation of Parkin-mediated mitophagy, through which damaged mitochondria are cleared more efficiently, and mitochondrial quality control is improved. This suggests that non-pharmacological strategies targeting Parkin-dependent mitophagy may represent a promising direction for the treatment of IRI related organ injury [214] (Table 2).

**Figure 2:**
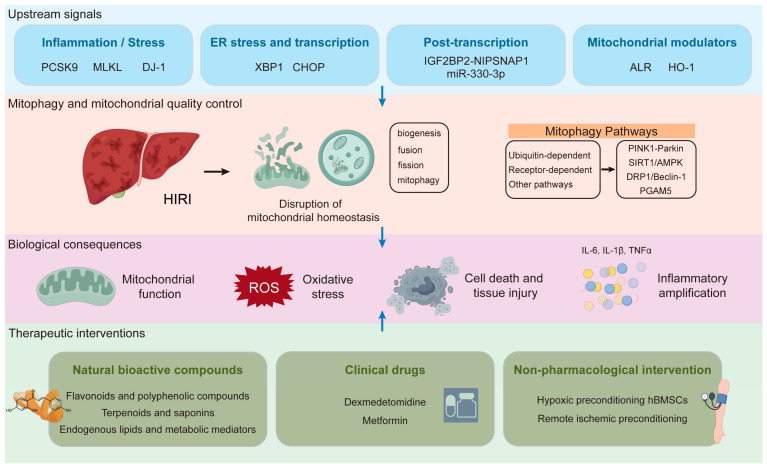
Layered framework of mitophagy regulation and therapeutic targeting in hepatic ischemia–reperfusion injury.

Taken together, whether through natural bioactive compounds, clinically used pharmacological agents, or non-pharmacological preconditioning approaches, a shared protective mechanism in HIRI lies in the remodeling of mitochondrial quality control, particularly at the levels of mitophagy, mitochondrial dynamics, mitochondrial biogenesis, and oxidative stress regulation. Notably, the role of mitophagy across different therapeutic strategies is not invariably one of simple enhancement; in some contexts, moderate suppression of aberrantly activated mitophagy may also confer protection. Future studies should therefore place greater emphasis on how distinct therapeutic interventions coordinately regulate the mitochondrial quality control network, thereby providing a more robust theoretical basis for precision targeted intervention in HIRI (Figure 2).

## 7. Discussion

HIRI, as one of the major determinants of patient prognosis, involves a constellation of complex biological mechanisms in which mitochondria play a central role. Mitochondria are not only the core of cellular energy metabolism, but also key regulatory hubs for stress responses, redox balance, and cell death. With the deepening investigation of mitochondrial dysfunction and mitophagy in HIRI, it has become increasingly clear that imbalance in mitochondrial quality control is closely associated with the severity of liver injury. Nevertheless, despite substantial progress in this field, many controversies remain, and major challenges continue to hinder clinical translation [215].

This review has examined in depth the role of mitochondria in HIRI, with particular emphasis on mitochondrial dysfunction and the dynamic changes in mitophagy during the ischemic and reperfusion phases. During ischemia, mitochondrial ATP production and oxidative phosphorylation decline markedly, forcing cells to shift toward anaerobic metabolism and thereby aggravating metabolic disturbance. During reperfusion, the restoration of oxygen supply exposes mitochondria to intense oxidative stress, resulting in a burst of ROS production and mitochondrial calcium overload, which ultimately exacerbates cellular injury [216]. In addition, the role of mitophagy in ischemia–reperfusion injury cannot be simply summarized as “more is protective.” When mitochondrial injury remains potentially reversible, moderate activation of mitophagy facilitates the removal of depolarized and dysfunctional mitochondria, limits sustained mitochondrial ROS generation, preserves intracellular homeostasis, and thereby attenuates hepatocellular injury [217]. However, when ischemia is prolonged or when oxidative stress and inflammation persist after reperfusion, leading to sustained opening of the mPTP and severe loss of mitochondrial membrane potential, mitophagic flux becomes insufficient to eliminate damaged mitochondria, thereby further aggravating hepatic injury. Thus, while appropriate mitophagy helps mitigate liver injury, both excessive and insufficient autophagic responses may worsen damage, underscoring the biphasic nature of mitophagy regulation. From a therapeutic perspective, this means that mitophagy should not be targeted through nonspecific enhancement alone. Excessive or prolonged mitophagy may deplete the functional mitochondrial pool and worsen ATP deficiency, whereas incomplete mitophagic flux may permit damaged mitochondria or mitophagic intermediates to accumulate. Therefore, the therapeutic goal should be to restore appropriate and complete mitophagic flux within a defined temporal and metabolic window rather than simply increasing mitophagy activation.

At a deeper level, mitophagy in HIRI should not be viewed as an isolated process but rather understood within the broader network of mitochondrial quality control [218]. Mitophagy, fission, fusion, and biogenesis are structurally and functionally interconnected, and together determine mitochondrial population homeostasis and the adaptive capacity of hepatocytes under stress [219]. Moderate mitochondrial fission facilitates the entry of damaged mitochondria into the clearance pathway, whereas excessive fission or insufficient fusion promotes mitochondrial fragmentation, loss of membrane potential, and increased Cytochrome C release [220]. Likewise, even if mitophagy is enhanced, mitochondrial homeostasis cannot be sustained when biogenesis remains inadequate. In other words, the critical issue in HIRI is not merely whether the mitophagy pathway is activated, but whether the mitochondrial quality control network remains functionally integrated.

Moreover, the reported findings regarding mitophagy in HIRI are not entirely consistent. First, the regulatory pathways governing mitophagy extend beyond the canonical PINK1/Parkin axis, and receptor-dependent and lipid-mediated mechanisms remain insufficiently characterized. The classical PINK1/Parkin pathway is generally considered to exert a relatively clear protective role in most studies, with its activation typically associated with enhanced clearance of damaged mitochondria, reduced hepatocyte apoptosis, and suppression of inflammasome activation. By contrast, conclusions regarding receptor-dependent mitophagy molecules such as FUNDC1 remain less consistent across studies. For example, ULK1 promotes mitophagy activation by phosphorylating FUNDC1 at Ser17. Conversely, Src and CK2 inhibit the interaction between FUNDC1 and LC3 through phosphorylation at Tyr18 and Ser13, thereby suppressing mitophagy [141,142]. Current understanding of cell type-specific differences in mitophagy also remains limited. Most studies have centered on hepatocytes, emphasizing the roles of mitophagy in sustaining energy metabolism, reducing ROS, and suppressing apoptosis. However, HIRI is not confined to hepatocytes alone, but also involves the coordinated participation of Kupffer cells, liver sinusoidal endothelial cells, neutrophils, and other cellular populations. In addition, different ischemia–reperfusion models vary substantially in ischemic duration, reperfusion conditions, and inflammatory background, all of which can influence the severity of mitochondrial dysfunction and the nature of the autophagic response. Accordingly, the regulatory network of mitophagy displays marked organ specificity, cell specificity, and dependence on injury timing. Current interpretations of the relevant pathways must therefore avoid oversimplification, and findings from other organ systems should not be mechanically extrapolated to the liver.

Despite these unresolved issues, mitophagy remains one of the most promising directions for translational research in HIRI. In clinical contexts such as liver transplantation, hepatectomy, and hemorrhagic shock, preconditioning and targeted interventions centered on mitochondrial quality control hold substantial promise. A variety of strategies-including natural bioactive compounds, exosome-based delivery systems, and remote ischemic preconditioning have already been shown to alleviate HIRI by enhancing protective mitophagy, improving mitochondrial membrane potential, and suppressing NLRP3 inflammasome activation [221,222]. Although these studies have revealed the therapeutic potential of mitophagy at the level of basic research, major obstacles remain before this mechanism can be translated into clinical therapy. First, HIRI occurs within a highly dynamic perioperative environment, making the timing of intervention critically important. At the same time, the complexity and dual nature of mitophagy make it a difficult therapeutic target. Excessive activation of mitophagy may promote cell death, whereas inhibition of mitophagy may result in the accumulation of damaged mitochondria [223]. Without precise identification of the optimal temporal window at different stages of injury, therapeutic efficacy may be diminished or even reversed. Thus, achieving fine-tuned regulation of mitophagy to maximize therapeutic benefit remains a central challenge in clinical translation.

Second, the safety and efficacy of mitochondria-targeted therapies require further validation. At present, many studies have focused on natural bioactive molecules or pharmacological agents that protect the liver by modulating mitophagy or mitochondrial function. However, these approaches remain at an early preclinical stage, with most available evidence derived from animal models and insufficient data regarding their long-term efficacy and safety. In addition, while current research has largely focused on the basic mechanisms of mitophagy and related drug interventions, systematic investigation of mitophagic alterations under specific pathological conditions remains lacking [224,225]. Future studies should therefore place greater emphasis on developing individualized therapeutic strategies based on the regulation of mitophagy in distinct disease backgrounds. With the continuing development of precision medicine, precise modulation of mitophagy is likely to become a future direction in the treatment of HIRI [226]. Advanced molecular biological approaches to dissect key regulatory factors and signaling pathways of mitophagy, combined with gene editing technologies, may provide more accurate options for individualized treatment. At the same time, drug targeting efficiency and bioavailability remain major barriers to clinical translation. How to deliver mitophagy-regulating agents effectively to the liver and achieve the desired therapeutic outcomes will require extensive basic research and clinical validation [227]. Therefore, the precise control of the timing and magnitude of mitophagy under different pathological conditions, together with the development of novel drug delivery systems to improve the efficiency and specificity of mitochondrial targeting, remains an urgent challenge if liver injury is to be minimized to the greatest extent possible [228].

Finally, several methodological and translational limitations of current HIRI research should be acknowledged. First, the evaluation of ex vivo mitochondrial function in HIRI models is highly dependent on pre-analytical and technical variables [229]. Isolated mitochondrial respiration can be influenced by tissue harvest time, cold ischemia duration, homogenization intensity, centrifugation conditions, buffer composition, osmolarity, pH, calcium chelation, and temperature [230]. Among these factors, buffer selection is particularly important because it may substantially affect mitochondrial yield, structural integrity, and respiratory performance [231]. Comparative evidence suggests that MOPS–sucrose buffer may be more suitable for isolating high-quality functional mitochondria from soft tissues such as liver and brain, whereas Tris–mannitol buffer may be more appropriate for hard tissues such as skeletal muscle and heart [232]. Therefore, studies assessing hepatic mitochondrial respiration should clearly report the isolation protocol and buffer composition, and should validate mitochondrial integrity rather than assuming that different isolation procedures are interchangeable. To improve methodological rigor, future studies should include quality-control parameters such as respiratory control ratio, citrate synthase activity, mitochondrial membrane integrity, Cytochrome C responsiveness, contamination by non-mitochondrial organelles, and the strategy used for data normalization [233,234]. Whenever possible, isolated mitochondrial assays should also be complemented by permeabilized liver tissue, high-resolution respirometry, in situ imaging, or spatial omics approaches, which may better preserve tissue architecture and cell-type heterogeneity [235]. Second, the translational relevance of current mechanistic evidence remains limited. Most studies on mitophagy in HIRI have been performed in rodent models [236]. Although these models are indispensable for pathway dissection and genetic manipulation, they do not fully reproduce human hepatic metabolic responses, organ size, lobular architecture, biliary anatomy, immune–vascular interactions, perioperative complexity, or the metabolic heterogeneity of transplant recipients. Accordingly, conclusions derived from rodent studies should not be directly extrapolated to clinical HIRI without cross-species validation. Future research should place greater emphasis on large mammalian models, particularly porcine or other livestock models, which more closely resemble human liver size, vascular anatomy, surgical handling, and tissue-specific mitochondrial energy dynamics [237,238]. In parallel, human-based experimental platforms, including discarded donor liver tissues, precision-cut liver slices, normothermic machine perfusion systems, organoids, and liver-on-chip models, may help bridge the gap between rodent mechanisms and clinical translation. Such approaches will be essential for determining whether mitophagy-targeted interventions are protective, neutral, or harmful in clinically relevant metabolic contexts.

## 8. Conclusions

In summary, current evidence clearly indicates that HIRI is not merely an isolated event of oxidative stress or inflammation but a complex pathological process in which mitochondrial injury and the disruption of mitochondrial quality control play fundamental roles. The importance of mitophagy in HIRI is increasingly supported by experimental evidence, and therapeutic strategies targeting this mechanism have shown considerable potential for clinical translation. Nevertheless, many challenges remain, particularly with respect to achieving precise regulation of mitophagy while avoiding its adverse effects. Future research should place greater emphasis on the complexity of the mitophagy regulatory network, further elucidate autophagic responses under diverse pathological backgrounds, and explore how modern precision medicine can be integrated to achieve individualized therapy. As the field continues to advance, mitophagy is expected to emerge as an important therapeutic target not only for HIRI, but also for other diseases associated with mitochondrial dysfunction, thereby offering new avenues for clinical intervention.

## Figures and Tables

**Table 1:** Key regulators of mitophagy in HIRI.

Factors	Expression Change	Influence Pathways	Role in HIRI	Effect of Modulation	Reference
PCSK9	Upregulate	PINK1/Parkin STING/NLRP3	Inhibits mitophagy; promotes inflammation	Inhibition restores mitophagy and alleviates HIRI	[172]
DJ-1	Downregulate	PINK1/Parkin	Restrains PINK1/ Parkin-mediated mitophagy	Deficiency enhances mitophagy and reduces injury	[173]
MLKL	Upregulate	PINK1/Parkin cGAS–STING	Suppresses mitophagy; activates cGAS–STING signaling	Deficiency enhances mitophagy and attenuates inflammation	[174]
XBP1	Upregulate	FOXO1/PINK1/Parkin	Inhibits FOXO1/PINK1/Parkin signaling	Deletion restores mitophagy and reduces hepatocyte injury	[176]
ALR	-	Mfn2/PINK1/ Parkin	Supports Mfn2-dependent mitophagy	Restoration promotes mitophagy and reduces apoptosis	[180]
NIPSNAP1	Downregulate	IGF2BP2/NIPSNAP1	Promotes mitophagy and mitochondrial homeostasis	Overexpression enhances mitophagy; knockdown suppresses it	[182]
CHOP	Upregulate	DRP1/Beclin-1	Impairs adaptive mitophagy under stress	Deficiency enhances mitophagy and reduces ROS/cell death	[184]
miR-330-3p	Downregulate	PGAM5/PINK1	Suppresses PGAM5-associated mitophagy	Downregulation enhances PGAM5-dependent mitophagy	[186]
HO-1	Upregulate	PGAM5	Maintains mitochondrial quality control	Activation promotes PGAM5-related mitochondrial protection	[187]

**Table 2:** Representative therapeutic interventions targeting mitophagy in HIRI.

Intervention	Category	Experimental Model	Molecular Targets	Effects	Reference
Tangeretin (TAN)	Polymethoxylated flavone	Mouse hepatic I/R model; hepatocytes	Nrf2/Keap1	Enhance mitophagy Reduce intracellular ferrous iron and LPO level	[197]
Pterostilbene (Pt)	Polyphenolic stilbene	C57BL/6 mouse hepatic I/R model; hepatocyte A/R model	PINK1/Parkin	Enhance mitophagy Alleviate apoptosis, mitochondrial membrane dysfunction and mtROS accumulation	[198]
Taxifolin (TAX)	Flavonoid	Mouse hepatic I/R model; primary hepatocytes	PINK1/Parkin	Enhance mitophagy Restore mitochondrial membrane potential Suppress pro-apoptotic proteins	[199]
Formononetin (FN)	Isoflavone	Rat hepatic I/R model	PHB2/PINK1/Parkin	Enhance mitophagy	[200]
Genipin	Terpenoid derivative	Mouse hepatic I/R model	SIRT1/AMPK	Suppress mitochondrial fission Upregulate mitochondrial biogenesis, mitophagy, and fusion proteins	[201]
Ginsenoside Rg1	Triterpenoid glycoside	Rat hepatic I/R model; hepatocyte H/R model	PINK1/Parkin	Suppresses mitophagy excessive activation Inhibit apoptosis Stabilize MMP	[202]
Resolvin D1 (RvD1)	Endogenous lipid mediator	Rat hepatic I/R model	TRX2/TXNIP	Restore mitochondrial function Reduce oxidative damage	[204]
25-Hydroxycholesterol (25-HC)	Endogenous oxysterol	Mouse hepatic I/R model	PINK1/Parkin	Enhance mitophagy Suppress NLRP3 inflammasome activation	[205]
Nicotinic acid (NA)	Vitamin B3 derivative	Mouse hepatic I/R model; primary hepatocytes	mPTP stabilization	Suppress inflammatory responses Alleviate hepatic oxidative stress	[206]
Dexmedetomidine (Dex)	α2-adrenergic receptor agonist	Two-week-old rat hepatic I/R model	SIRT3/NLRP3	Enhance mitophagy Suppress inflammatory responses	[208]
Met-Exo	Drug-loaded exosome strategy	Miniature pig liver IRI model	AMPK/SIRT1	Reduce ROS accumulation Suppress pyroptosis Reduce mitochondrial injury	[209]
Hypoxic preconditioning hBMSCs	Non-pharmacological strategy	hBMSCs; hepatocytes; liver graft I/R model	-	Improve mitochondrial quality Promote high-quality mitochondria transfer	[212]
RIPC	Non-pharmacological strategy	Mouse hepatic I/R model; Parkin-deficient mouse model	PINK1/Parkin	Enhance mitophagy Suppress inflammatory responses	[214]

## Data Availability

No new data were created or analyzed in this study.

## Abbreviations

The following abbreviations are used in this manuscript:

2-ME2	2-Methoxyestradiol
3-MA	3-Methyladenine
25-HC	25-Hydroxycholesterol
ADSC-Exo	Adipose-derived stem cell exosomes
ALR	Augmenter of liver regeneration
ALT	Alanine aminotransferase
AMBRA1	Activating molecule in Beclin 1-regulated autophagy 1
AMPK	AMP-activated protein kinase
AST	Aspartate aminotransferase
ATP	Adenosine triphosphate
cGAS	Cyclic GMP-AMP synthase
CHOP	CCAAT/enhancer-binding protein homologous protein
DAMPs	Damage-associated molecular patterns
Dex	Dexmedetomidine
DJ-1	Parkinsonism-associated deglycase
eIF2α	Eukaryotic translation initiation factor 2 alpha
eNOS	Endothelial nitric oxide synthase
ER	Endoplasmic reticulum
FN	Formononetin
FUNDC1	FUN14 domain-containing protein 1
hBMSCs	Human bone marrow mesenchymal stem cells
HIRI	Hepatic ischemia–reperfusion injury
HO-1	Heme oxygenase 1
HSR	Hemorrhagic shock resuscitation
IGF2BP2	Insulin-like growth factor 2 mRNA-binding protein 2
LAMP2	Lysosome-associated membrane protein 2
LC3	Microtubule-associated protein 1 light chain 3
LDH	Lactate dehydrogenase
LIR	LC3-interacting region
